# Supplementary material for: Differential Effects of Cytokine Versus Hypoxic Preconditioning of Human Mesenchymal Stromal Cells in Pulmonary Sepsis Induced by Antimicrobial-Resistant Klebsiella pneumoniae
Source: Pharmaceuticals (Basel). 2023 Jan 19;16(2):149. doi: 10.3390/ph16020149 (PMC9961251; doi:10.3390/ph16020149)
Supplement: Supplementary file 1 [file pharmaceuticals-16-00149-s001.zip › pharmaceuticals-2059590-supplementary.pdf]

Supplementary Materials

Differential Effects of Cytokine Versus Hypoxic Preconditioning of Human Mesenchymal Stromal Cells in Pulmonary Sepsis Induced by Antimicrobial-Resistant *Klebsiella pneumoniae*

Declan Byrnes [1,2], Claire H. Masterson [1,2,*], Jack Brady [1,2], Senthilkumar Alagesan [1,2], Hector E. Gonzalez [1,2], Sean D. McCarthy [1,2], Juan Fandiño [1,2], Daniel P. O'Toole [1,2], and John G. Laffey [1,2,3]

[1] Anaesthesia, School of Medicine, University of Galway, H91 TK33 Galway, Ireland
[2] Regenerative Medicine Institute (REMEDI) at CÚRAM Centre for Research in Medical Devices, Biomedical Sciences Building, University of Galway, H91 TK33 Galway, Ireland
[3] Department of Anaesthesia, Galway University Hospitals, SAOLTA University Health Group, H91 YR71 Galway, Ireland
* Correspondence: claire.masterson@universityofgalway.ie; Tel.: +353-91-495909

Supplementary Figures

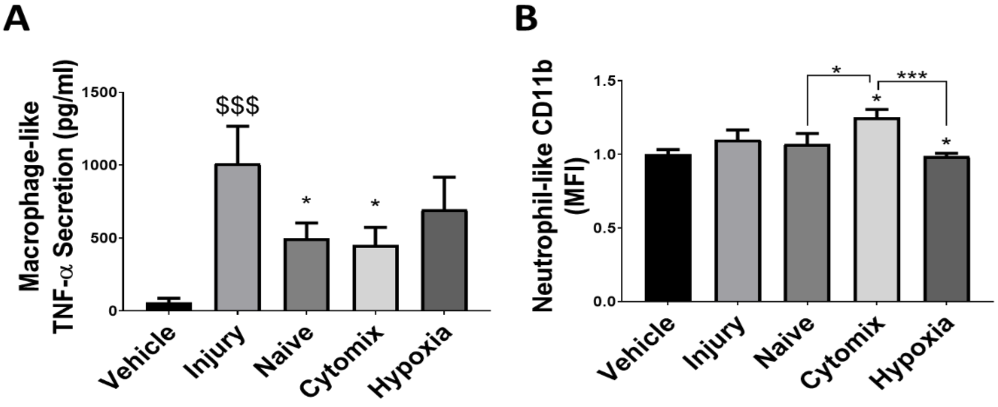

Figure S1. Terminally differentiated THP-1 (A) and HL-60 (B) to macrophages and neutrophils respectively. Macrophage-like cells exposed to an LPS injury concurrent to naïve and cytomix MSC-CM show significantly reduced TNF-α secretion compared to injury control (A). Neutrophil-like cells exposed to an LPS injury concurrent to cytomix MSC-CM show an enhanced CD11b expression while hypoxia MSC-CM significantly reduce the expression (B). .

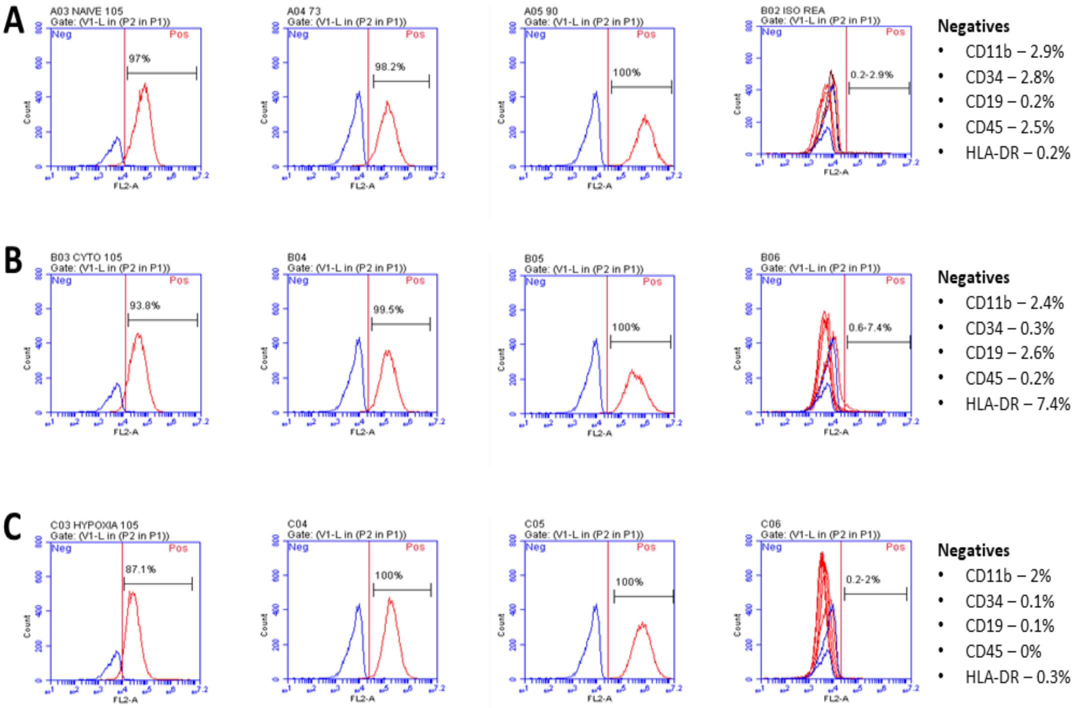

Figure S2. Characterisation of cryopreserved naïve (A), cytomix (B), and hypoxia (C) UC-MSCs for the expression of the positive markers CD105, CD73, and CD90 and the negative markers CD11b, CD34, CD19, CD45, and HLA-DR. Naïve MSCs maintained high expression of the positive markers and low expression of the negative markers after cryopreservation (A). Cytomix licensing of the cells had slightly reduced CD105 expression (93.8% positive) while increased expression of HLA-DR (7.1%) (B). Hypoxia culturing eroded the expression of CD105 (87.1% positive) but strongly preserved the lack of negative marker expression (C).   .

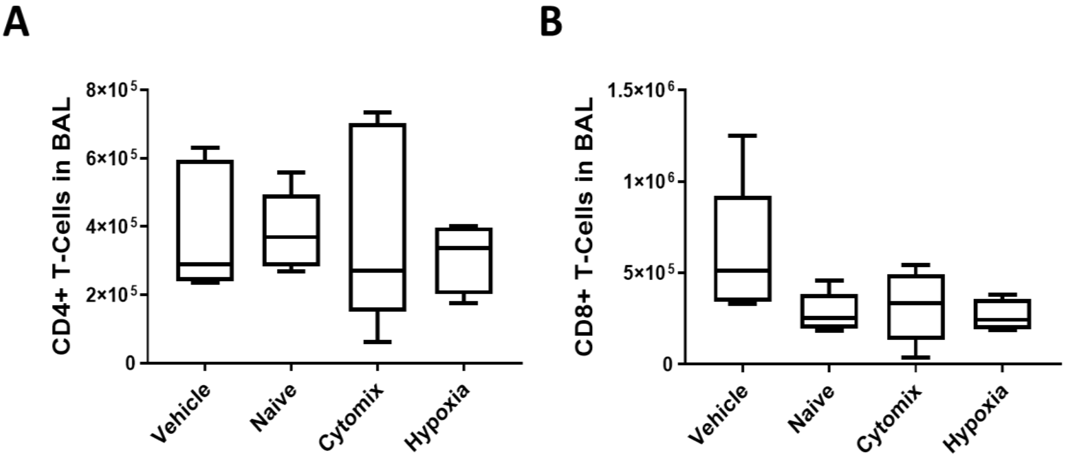

Figure S3. Total live CD3+, CD4+ T helper cells (A) and CD8+ cytotoxic T cells (B) in the BAL, as analysed by flow cytometry. BAL = bronchoalveolar lavage. Box plots and whiskers represent minimum, first quartile, median, third quartile, and maximum. * = P≤0.05 wrt vehicle control. N= 8-12.

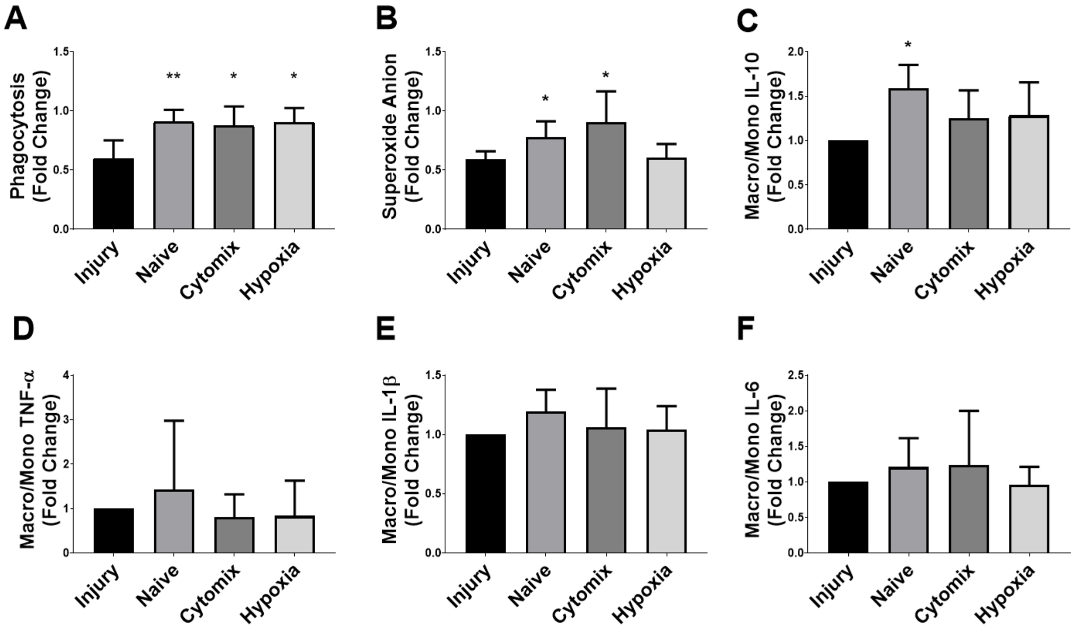

Figure S4. Naïve and cytomix licensed MSC administration significantly improved macrophage/monocyte cell function after an ex vivo LPS injury (A & B). Naïve MSCs improved cytokine secretion of these cells on average in response to an LPS stimulus (C – F), only IL-10 secretion reached significance (E). All data point symbols represent individual data points, columns represent mean. Error bars represent SD. *=P≤0.05 wrt injury control. .

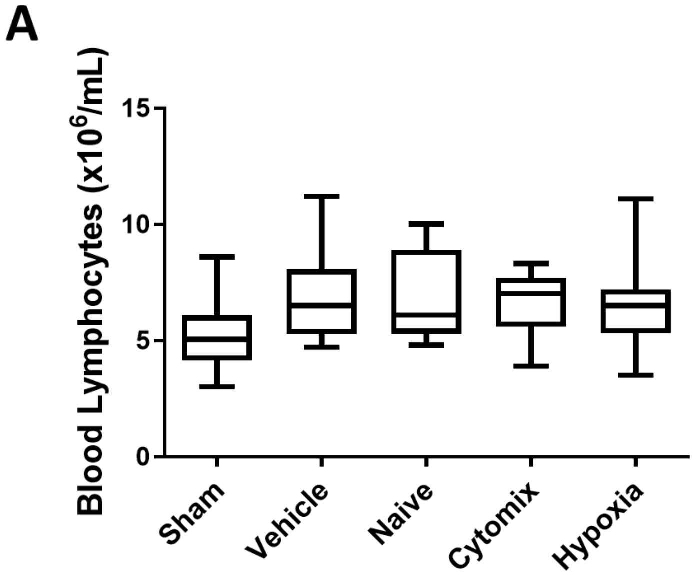

Figure S5. Total blood lymphocytes, as analysed by a haemoanalyser. Box plots and whiskers represent minimum, first quartile, median, third quartile, and maximum. * = P≤0.05 wrt vehicle control. $ = P ≤0.05 wrt sham group. N= 8-12.

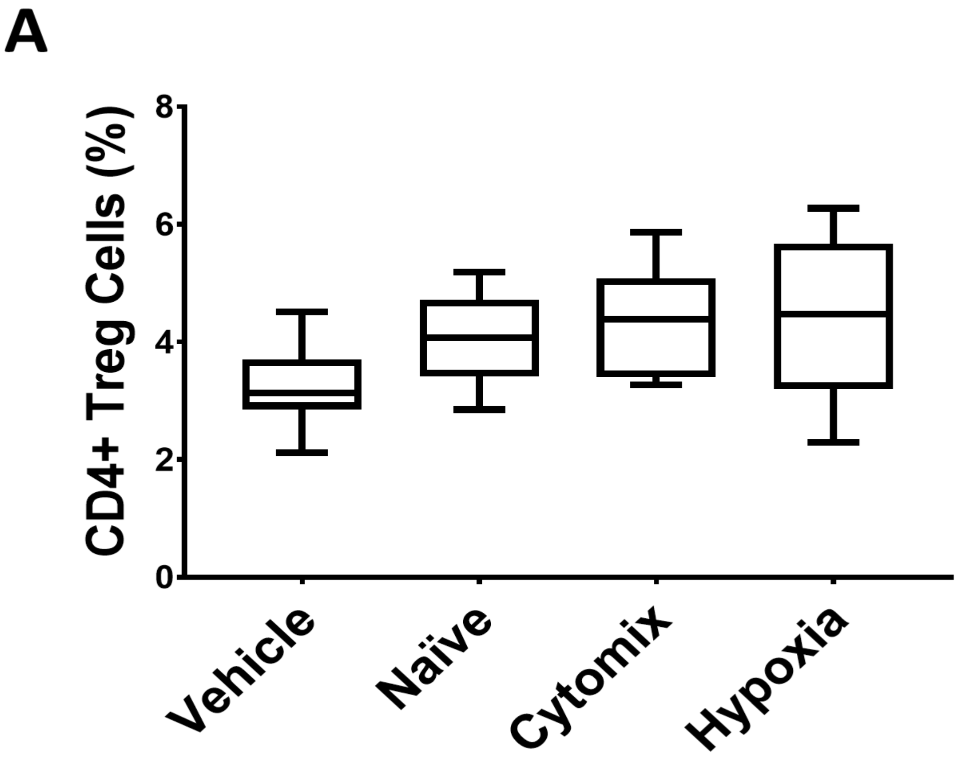

Figure S6. Percentage of CD3+, CD4+ regulatory T cells (CD25+, Foxp3+) in the PBMC population, as analysed by a flow cytometry. Box plots and whiskers represent minimum, first quartile, median, third quartile, and maximum. * = P≤0.05 wrt vehicle control. N= 8-12.

Table S1. Flow cytometry-based surface/intra-cellular marker staining combinations for defined immune cell sub-populations from PBMCs.

Immune Cell Type	Staining Combination
Monocytes	Live, single, $CD3^-CD45^+CD11bc^+$
Classical monocytes	Live, single, $CD3^-CD45^+CD11bc^+CD43^-$
Non-classical monocytes	Live, single, $CD3^-CD45^+CD11bc^+CD43^+$
$CD4^+$T-cells	Live, single, $CD3^+$ $CD4^+$
$CD8^+$ T-cells	Live, single, $CD3^+$ $CD8^+$
Regulatory T-cells	Live, single, $CD3^+CD25^+FOXP3^+$

Monocytes:

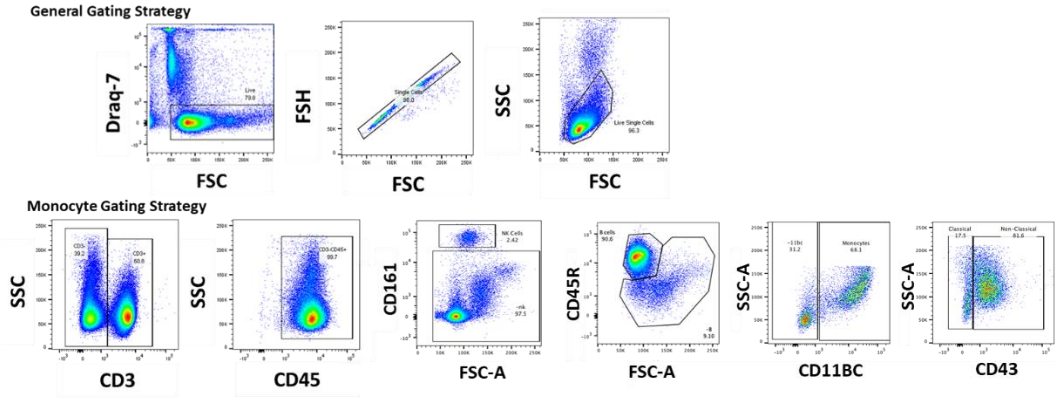

T-cells & T-regs:

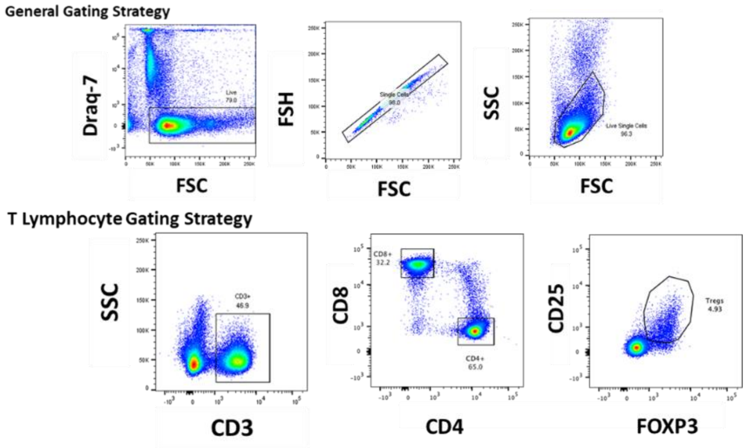

Figure S7. Gating Strategy for monocyte and T-cell sub-sets from PBMCs.

Supplementary Materials and Methods

THP-1 differentiation and LPS injury

For macrophage differentiation, THP-1's were plated in a 96-well plate at 1x106 cells/mL in the presence of 25nM phorbol 12-myristate 13-acetate (PMA) for 48 hours to allow for attachment to occur and adaptation of macrophage-like morphology. The cells were then washed and allowed to rest for 24 hours. Terminally differentiated THP-1's were incubated with MSC-CM and 100 ng/mL K. pneumoniae LPS for 24 hours. After the incubation, the cells were washed and injured again with 100 ng/mL for 4 hours before media collection and subsequent TNF-α ELISA.

HL-60 differentiation and LPS injury

Differentiation of HL-60s to neutrophil-like cells was with the addition of 1.5% DMSO (Sigma-Aldrich) into standard cell line media and the cells were left in culture for 6 days with a media change occurring after 3 days in culture. Terminally differentiated HL-60's were incubated with 3.5 μg/mL K. pneumoniae LPS for 24 hours before the addition of MSC-CM and incubated again for 24 hours. HL-60s were washed and stained with the anti-human marker CD11b (PerCP-vio700) and their median fluorescent intensity (MFI) was recorded on the Accuri C6 flow cytometer. Appropriate isotype controls were used during the analysis.

Human Umbilical-cord derived MSCs characterisation

Flow cytometry analysis was done on the cryopreserved UC-MSCs utilising the Accuri C6 Plus flow cytometer. MSCs were suspended at 2x106 per mL and 100 μL of the suspension was placed per well of a 96-well plate. The wells were individually stained with R-phycoerythrin (PE) labelled anti-human antibody in FACS buffer (DPBS containing 5% FBS) for the positive markers CD105, CD73, and CD90 (BD Biosciences) and the negative markers CD45, CD34, CD11b, CD19, and HLA-DR (ImmunoTools, GmbH, Friesoythe, Germany) with the appropriate isotype controls. Live/Dead™ Far Red was used in tandem with the PE antibodies to exclude any dead cells. Samples were kept on ice, in the dark, for 30 minutes after the addition of the dye and subsequently washed before being brought to the Accuri to measure the percentage positive surface marker.
